# Automated Malaria Ring Form Classification in Blood Smear Images Using Ensemble Parallel Neural Networks

**DOI:** 10.3390/jimaging12030127

**Published:** 2026-03-12

**Authors:** Pongphan Pongpanitanont, Naparat Suttidate, Manit Nuinoon, Natthida Khampeeramao, Sakhone Laymanivong, Penchom Janwan

**Affiliations:** 1 Health Sciences (International Program), College of Graduate Studies, Walailak University, Nakhon Si Thammarat 80160, Thailand; pongphan.po@mail.wu.ac.th (P.P.); naparat.st@mail.wu.ac.th (N.S.); natthida.kh@mail.wu.ac.th (N.K.); 2 Akkhraratchakumari Veterinary College, Walailak University, Nakhon Si Thammarat 80160, Thailand; 3 Centre for One Health, Walailak University, Nakhon Si Thammarat 80160, Thailand; 4 Department of Medical Technology, School of Allied Health Sciences, Walailak University, Nakhon Si Thammarat 80160, Thailand; manit.nu@wu.ac.th; 5 Hematology and Transfusion Science Research Center, Walailak University, Nakhon Si Thammarat 80160, Thailand; 6 Centre of Malariology, Parasitology and Entomology, Ministry of Health, Vientiane P.O. Box 0100, Laos; sakhone07@gmail.com

**Keywords:** malaria, blood smear image, deep learning, image classification, neural networks

## Abstract

Manual microscopy for malaria diagnosis is labor-intensive and prone to inter-observer variability. This study presents an automated binary classification approach for detecting malaria ring-form infections in thin blood smear single-cell images using a parallel neural network framework. Utilizing a balanced Kaggle dataset of 27,558 erythrocyte crops, images were standardized to 128 × 128 pixels and subjected to on-the-fly augmentation. The proposed architecture employs a dual-branch fusion strategy, integrating a convolutional neural network for local morphological feature extraction with a multi-head self-attention branch to capture global spatial relationships. Performance was rigorously evaluated using 10-fold stratified cross-validation and an independent 10% hold-out test set. Results demonstrated high-level discrimination, with all models achieving an ROC–AUC of approximately 0.99. The primary model (Model#1) attained a peak mean accuracy of 0.9567 during cross-validation and 0.97 accuracy (macro F1-score: 0.97) on the independent test set. In contrast, increasing architectural complexity in Model#3 led to a performance decline (0.95 accuracy) due to higher false-positive rates. These findings suggest that moderate-capacity feature fusion, combining convolutional descriptors with attention-based aggregation, provides a robust and generalizable solution for automated malaria screening without the risks associated with over-parameterization. Despite a strong performance, immediate clinical use remains limited because the model was developed on pre-segmented single-cell images, and external validation is still required before routine implementation.

## 1. Introduction

Malaria remains a life-threatening parasitic disease and a persistent global health concern, with an ongoing burden of severe morbidity and mortality. Human malaria is caused by five *Plasmodium* species, most prominently *Plasmodium falciparum* and *Plasmodium vivax*; *P. falciparum* is associated with the highest case fatality and is predominant in Africa [1]. In routine practice, the reference diagnostic workflow is the microscopic examination of Giemsa-stained blood smears by trained technicians. While effective, manual microscopy is labor-intensive and time-consuming, commonly requiring 10 to 15 min per slide. Furthermore, this process may be affected by parasite sparsity, observer fatigue, and inter-reader variability, particularly in high-volume settings with limited expert availability [2]. These constraints motivate automated approaches that can assist diagnosticians by improving throughput and standardizing interpretation. Deep learning-based image analysis has emerged as a leading approach for computer-aided malaria diagnosis from blood smear images. This technology can learn discriminative representations directly from pixels and can be regularized to handle nuisance variation in staining, illumination, and background artifacts [3].

Historically, convolutional neural networks (CNNs) have constituted the backbone of malaria cell-image classification. Early work established that CNNs can outperform classical pipelines relying on hand-crafted features, demonstrating that task-driven feature learning is well suited to the fine-grained morphological and textural cues present in thin-smear microscopy [4]. Building on this foundation, subsequent studies have explored both compact, custom CNNs optimized for malaria datasets and deeper architectures pretrained on large natural-image corpora, such as ImageNet. These include the VGG, ResNet, Inception, Xception, and EfficientNet families [5]. In general, transfer learning reduces the data requirement for training deep models from scratch and enables rapid convergence while still achieving strong performance after task-specific fine-tuning. Beyond canonical backbones, malaria studies increasingly incorporate parameter-efficient variants to balance accuracy with deployability. EfficientNet-family models and lightweight MobileNet variants have been reported to achieve competitive results while reducing memory footprint and inference cost, which is relevant to point-of-care screening and mobile deployment [2]. Capsule-inspired hybrids have also been explored to improve representational richness and interpretability by explicitly modeling part-whole relationships in parasite morphology [6].

To mitigate the limitation of purely local receptive fields in standard CNNs and to better emphasize parasite-relevant regions, recent work has introduced attention mechanisms as add-on modules within CNN pipelines. Spatial and channel attention can reweight intermediate features, helping models focus on diagnostically salient intra-erythrocytic structures while suppressing background artifacts and staining debris [2]. Such modules are particularly relevant when discriminative cues are subtle and occupy a small fraction of the cell crop, as is common for ring-form infections. Vision transformers (ViTs) and hybrid CNN-transformer architectures have also been investigated for malaria microscopy, leveraging self-attention to capture longer-range dependencies and patch-level relationships. Reports indicate that transformer-based models can match or exceed CNN performance in multi-class recognition settings when training data are sufficiently large and diverse, and they can provide attention maps that support qualitative interpretability of model focus [7,8]. In practice, transformer components are often integrated with convolutional stems or multi-scale designs to preserve local texture sensitivity while adding global context aggregation.

Ensemble learning is frequently used to improve robustness by combining the outputs of multiple models through voting or probability averaging. Such ensembles can modestly improve sensitivity and reduce variance relative to single models, even at the cost of increased computation [9]. Hybrid pipelines have also been proposed, including CNN feature extraction followed by classical classifiers such as SVM, or two-stage workflows that couple candidate selection with confirmatory deep classification [3]. These strategies are often motivated by the need to control false negatives in screening contexts while maintaining acceptable specificity. Clinical translation often requires extending from single-cell classification to slide-level analysis, where the practical objective is to locate rare infected cells across large fields of view. Accordingly, object detection formulations such as Faster R-CNN and YOLO-family detectors have been applied to localize and classify parasites within smear images [10,11]. Detection-driven approaches are central to operational deployment because they enable parasite localization, parasitemia estimation, and downstream quality control on full-field images.

Across curated benchmark datasets, many deep learning models report high accuracy with strong precision, recall, and F1-scores, including ROC-AUC values near 0.99 [4,9]. However, reported performance is sensitive to the experimental protocol, especially the split strategy and any potential leakage across correlated samples. Where metadata permit, patient-level or slide-level partitioning typically yields more conservative estimates than random image-level splitting when multiple images originate from the same patient or slide [12]. Consequently, careful validation design and transparent reporting of splitting, augmentation, and statistical uncertainty remain essential for interpreting comparative results and for estimating generalization under real deployment conditions. Building upon these developments, this study proposes an automated approach using ensemble parallel neural networks to optimize the classification of malaria ring-forms in blood smear images.

## 2. Literature Review

Convolutional neural networks (CNNs) have been established as a foundational approach for malaria cell-image classification, delivering major gains in diagnostic accuracy and workflow efficiency compared with manual microscopy. As a historical baseline in automated malaria diagnosis, CNNs have consistently demonstrated strong performance on infected vs. uninfected cell recognition tasks, with well-known architectures such as AlexNet and VGG19 achieving high classification metrics [13]. This progress directly addresses key limitations of conventional microscopy—namely that it is labor-intensive, time-consuming, and susceptible to inter-observer variability and human error [14]. In comparative studies, CNN-based approaches have also outperformed traditional integrated learning pipelines, reaching accuracies as high as 98.29% for malaria cell recognition. Building on this baseline, transfer learning has further strengthened CNN performance and practicality by leveraging pre-trained models to reduce training time while improving generalization, particularly when malaria datasets are limited in size. By transferring feature representations learned from large-scale image corpora, CNN classifiers can more reliably identify malaria patterns and achieve robust test performance; for instance, CNNs combined with transfer learning have reported testing accuracy around 96.94%, highlighting their stability across evaluation settings [15]. Alongside these improvements, lightweight and computationally efficient CNN variants have been developed to enable deployment in real clinical and point-of-care contexts, where hardware and energy constraints are common [16]. Such lightweight architectures can maintain high accuracy (approximately 96% in some reports) while reducing complexity and inference cost, sometimes outperforming heavier networks like VGG-19 and Inception v3 in efficiency without sacrificing diagnostic value. Reliability has also been extended through adversarially robust CNN designs, which aim to reduce sensitivity to small input perturbations and improve trustworthiness in automated diagnosis [17]. Despite these advances, important challenges remain—particularly dataset bias (e.g., lab/site-specific staining and imaging differences) and limited interpretability of learned features—both of which can hinder safe generalization to real-world settings. Future work is therefore well-motivated to emphasize multi-class classification (e.g., parasite stages/species), model optimization for edge deployment, and rigorous clinical validation to ensure robust performance across diverse populations and acquisition conditions. Overall, CNNs remain a core methodological foundation for malaria cell-image classification, and the continuing integration of transfer learning and lightweight architectures is steadily improving both performance and deployability, with strong potential to enhance diagnostic speed, consistency, and downstream patient outcomes in global health applications [18].

The integration of attention modules and transformer-based designs in medical imaging has substantially improved a model’s capacity to capture global context and long-range spatial dependencies—capabilities that are particularly important for detecting subtle or spatially dispersed parasite regions that may be missed when relying only on localized cues. In contrast to traditional CNNs, which are constrained by inherently local receptive fields, transformers use self-attention to model relationships across distant image regions, making them increasingly attractive for medical imaging applications [19,20]. This advantage has been especially evident in segmentation, where accurate delineation often depends on understanding global structure and long-range context; transformer-based and attention-driven segmentation approaches have reported strong performance by explicitly modeling these dependencies [21]. At the same time, hybrid CNN–transformer architectures have become prominent because they combine CNN strengths in extracting fine-grained local textures with transformer strengths in capturing global semantics, frequently yielding superior performance across diverse medical imaging tasks [22,23]. For example, CNN–transformer hybrid U-shaped designs incorporate convolutional blocks with attention mechanisms to jointly encode local and global information while managing computational cost, and contextual attention networks further enhance boundary clarity by blending long-range dependency modeling with localized semantic refinement. Beyond segmentation, clinical translation has also benefited from robust detection pipelines: ensemble methods, hybrid workflows, and established object-detection frameworks such as Faster R-CNN and YOLO have been adapted to medical imaging to support slide-level localization and clinically meaningful outputs like parasitemia estimation [24]. Recent transformer-based detection strategies have likewise been proposed for efficient malaria detection across variable parasite sizes, emphasizing both precision and computational efficiency for time-sensitive diagnosis, and transformer-driven approaches in parasitic egg detection have shown notable accuracy gains that may reduce the burden of manual microscopy for laboratory personnel [25]. Despite these advantages, transformer and hybrid approaches can face practical barriers, including the need for large-scale annotated datasets and substantial compute, which may restrict deployment in resource-limited settings [26]. Nevertheless, continued work on lightweight transformer variants and compute-aware hybrid designs is progressively mitigating these constraints and improving accessibility for real-world clinical use.

The National Library of Medicine (NLM) malaria dataset has been widely leveraged as a benchmark resource for accelerating automated malaria detection research, particularly through deep learning–based image classification pipelines. Prior studies consistently report strong discriminatory performance on this dataset using CNNs, transfer learning, hybrid deep architectures, and complementary feature-engineering strategies, underscoring the dataset’s utility for rapid methodological iteration and comparative evaluation. Transfer learning, in particular, has been repeatedly shown to improve classification accuracy while reducing training time by adapting representations learned from large-scale pretraining to malaria microscopy images—an advantage that is especially relevant for settings with limited computational capacity. Beyond transfer learning, standard CNNs and lightweight architectures (e.g., ShuffleNet) have achieved very high reported accuracies, with some work indicating test performance approaching 99.77%, while hybrid frameworks that combine deep learning with additional techniques have been proposed to enhance diagnostic accuracy and practical accessibility [27,28]. Comparative assessments across architectures further suggest meaningful performance variability, with models such as DenseNet161 and ResNet18 frequently evaluated and DenseNet161 reported as among the strongest performers in at least one study [29].

Despite these methodological gains, the translation of high benchmark performance into reliable real-world deployment remains constrained by several persistent challenges. A central concern involves bias and calibration: models trained on datasets with class imbalance, limited acquisition heterogeneity, or non-representative sampling may demonstrate inflated in-dataset metrics yet fail to generalize equitably across populations, laboratories, microscopes, staining protocols, and geographic contexts, thereby motivating stronger attention to calibration and interpretability during validation [30]. Relatedly, limited data diversity and annotation variability can reduce diagnostic robustness, prompting the use of methods such as GAN-based augmentation to broaden the effective support of training distributions and mitigate overfitting to narrow visual patterns. In parallel, hardware-aware optimization remains a practical bottleneck for implementation in resource-constrained environments, where memory, compute, battery constraints, and on-device latency necessitate efficient architectures, compression, and deployment-oriented design choices rather than accuracy-only optimization.

Consequently, current evidence indicates that future progress should focus less on incremental benchmark gains and more on deployment-aligned reliability. Explainable AI is increasingly positioned as a prerequisite for clinical adoption, as interpretability mechanisms can support clinician trust, enable error analysis, and clarify decision rationales under domain shift. In addition, collaborative global data-sharing initiatives and the construction of more diverse, standardized datasets are essential for reducing regional bias and improving generalization across heterogeneous care settings. Finally, ensuring scalability and robustness—particularly under data scarcity, instability in acquisition conditions, and operational constraints—remains critical for sustainable real-world use, emphasizing the need for rigorous external validation and deployment-centric evaluation protocols [31]. Overall, while the NLM malaria dataset has enabled substantial advances in automated malaria detection research, addressing bias, calibration, data diversity, and hardware-aware optimization is pivotal for successful translation into equitable and effective diagnostic systems.

In light of the strong but protocol-sensitive, performance reported across CNN, transfer-learning, and detection-driven malaria pipelines, our work aims to advance cell-level malaria screening by introducing an automated ensemble parallel neural-network framework that strengthens discrimination of malaria ring-forms in thin blood-smear images. Specifically, we target improved robustness by combining complementary feature pathways (rather than relying on a single backbone), with the goal of achieving a more stable sensitivity–specificity trade-off under realistic evaluation settings where splitting strategy, correlated samples, and potential leakage can materially affect reported accuracy. Building on prior evidence that model performance and deployment value depend on both validation rigor and operational error profiles, our approach is positioned as a practical step toward dependable, high-throughput malaria classification for downstream clinical workflows.

## 3. Materials and Methods

### 3.1. Dataset

**Figure 1:**
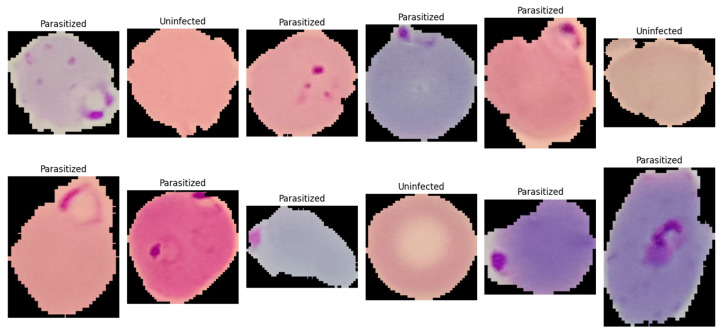
A single-cell image showing normal red blood cells (uninfected) and ring-form infected red blood cells (parasitized).

This study utilized the publicly available malaria thin blood smear dataset entitled “Cell Images for Detecting Malaria,” sourced from a Kaggle repository (Kaggle repository; available at: https://www.kaggle.com/datasets/iarunava/cell-images-for-detecting-malaria (accessed on 5 January 2026)) and originally developed by the National Institutes of Health (NIH) at the NLM. The dataset consisted of 27,558 pre-segmented single-cell crops acquired from thin blood smear microscopy and stored as RGB PNG images (Figure 1). The provided data were organized in a folder-per-class structure, namely Parasitized/and Uninfected/, enabling direct label assignment based on directory membership. Each sample represented an isolated erythrocyte (or a small erythrocyte-centered field) that had been cropped from a larger smear image during dataset curation, such that the classification task focused on identifying infection-related visual patterns at the cell level rather than performing whole-slide detection. Figure 1 is a representative visualization of the NIH-NLM/Kaggle single-cell crops used for binary classification. The present study does not perform object detection on full smear fields; consequently, bounding box annotations are not applicable as this study focuses strictly on image-level classification. Any image processing described in this work is limited to classification-oriented preprocessing (resizing, normalization, and rotation) applied before model inference.

The dataset constituted a balanced binary classification task with two categories: (i) Parasitized, referring to erythrocytes exhibiting visible *Plasmodium* infection signatures, and (ii) Uninfected, referring to erythrocytes without parasites. Each class contained 13,779 images, eliminating majority-class bias and simplifying interpretation of threshold-based metrics such as accuracy and F1-score. The dataset exhibited typical thin blood smear variability from slide preparation and imaging conditions, including variations in cell orientation, staining intensity, color tone, illumination uniformity, and background artifacts such as staining debris and boundary structures. Parasitized samples contained localized and subtle discriminative features, including small intra-erythrocytic structures and texture changes indicative of parasite presence, making the dataset suitable for evaluating architectures capable of capturing fine-grained local patterns. For reproducibility, images were ingested by enumerating file paths from both directories and assigning binary labels (1 = Parasitized, 0 = Uninfected). All images were processed as three-channel inputs, with downstream preprocessing standardizing resolution and intensity range (described in Section 3.3.3).

### 3.2. Data Partitioning and Validation Protocol

To obtain an unbiased estimate of generalization performance, all splitting procedures were performed using stratified sampling at the image level, such that the proportion of parasitized and uninfected images was preserved in every subset. Because subject- or slide-level identifiers were not available in the dataset, partitioning was conducted at the individual image granularity.

#### 3.2.1. Independent Stratified Hold-Out Test Split

Prior to any model development, a single stratified hold-out split was applied to separate an independent test set that remained completely untouched during model selection and hyperparameter tuning. Using a fixed random seed, the full dataset (27,558 images) was divided into the following:

- Training pool: 90% (24,802 images);
- Test set: 10% (2756 images).

Class stratification ensured that the test set contained exactly 1378 Parasitized and 1378 Uninfected images, maintaining the balanced class prior of the original dataset. The test set was used only once, after model development, to report final performance.

#### 3.2.2. Stratified 10-Fold Cross-Validation Within the Training Pool

Model selection and robustness assessment were conducted exclusively within the training pool by performing 10-fold stratified cross-validation. Specifically, the training pool was partitioned into 10 disjoint folds using stratified sampling. Across folds, the fold sizes were approximately equal, i.e., each validation fold contained about 2480–2481 images (≈1240 per class), and the corresponding training portion contained approximately 22,321–22,322 images.

For each fold k ∈ {1, …, 10}, the following protocol was applied:

- The *k*-th fold was designated as the validation subset, and the remaining nine folds were combined as the fold-specific training subset.
- A new instance of the network was initialized and compiled for the fold to prevent information carryover between folds.
- Data augmentation was applied only to the fold-specific training subset, whereas validation images were evaluated using preprocessing only (i.e., without stochastic augmentation).
- Model checkpointing and early stopping were driven by fold validation performance (monitored via validation area under the curve (AUC)), and the best checkpoint per fold was retained for metric computation.
- Fold-level predictions were generated for the validation fold, and fold-specific metrics were computed; subsequently, results were aggregated as mean ± standard deviation across folds.

This cross-validation design provided an estimate of model stability across different stratified partitions while preventing leakage from the held-out test set.

#### 3.2.3. Final Stratified Validation Split for Test Reporting

After cross-validation, a final training run was performed to produce a single model for reporting on the held-out test set. The training pool (24,802 images) was again partitioned using a stratified split into the following:

- Final training subset: 90% of the training pool (≈22,322 images);
- Validation subset: 10% of the training pool (≈2480 images).

This resulted in an overall approximate dataset allocation of 81%/9%/10% for train/validation/test, respectively, relative to the full dataset. During this final run, model selection (checkpointing) was performed using the validation subset, and the selected checkpoint was subsequently evaluated on the independent test set.

#### 3.2.4. Reproducibility Controls

To reduce run-to-run variability and improve reproducibility, a fixed seed was used to control pseudorandom behavior in Python (version 3.12.12), NumPy (version 2.0.2), and TensorFlow (version 2.19.0), and the same seed was consistently applied to all stratified splitting operations (hold-out split, cross-validation partitioning, and final train/validation split).

### 3.3. Preprocessing and Data Augmentation

All images were processed using a TensorFlow tf.data input pipeline to ensure reproducible and efficient streaming from disk to the GPU. Image file paths and binary class labels were first enumerated from the class directories and then converted into a tf.data.Dataset. The pipeline was implemented as a sequence of deterministic preprocessing transformations followed by stochastic augmentation operations applied only during training.

#### 3.3.1. Image Decoding and Tensor Formatting

Each sample was read from storage using tf.io.read_file, after which PNG decoding was performed with tf.image.decode_png to obtain a dense image tensor with three channels (RGB). Channel enforcement (channels = 3) was applied to avoid inconsistencies from potential grayscale files and to guarantee a fixed input tensor shape for the model.

#### 3.3.2. Spatial Resizing and Shape Standardization

To enforce a constant input resolution across the dataset and to meet the model input specification, each decoded image was resized to 128 × 128 pixels using tf.image.resize. This step ensured that both convolutional feature extractors and token/attention branches received inputs with identical spatial dimensions. The resulting tensor shape was standardized to (128, 128, 3).

#### 3.3.3. Type Casting and Intensity Normalization

After resizing, tensors were converted to floating-point representation (32-bit) and normalized to the range [0, 1] by division by 255. This normalization was used to reduce scale variability across samples and to stabilize optimization by keeping input magnitudes within a bounded interval. Labels were retained as scalar binary values compatible with the binary cross-entropy objective.

#### 3.3.4. On-the-Fly Data Augmentation (Training Only)

To improve generalization under realistic acquisition variation, stochastic augmentation was applied online during training and disabled during validation and test evaluation. Augmentations were applied on-the-fly so that each epoch could observe a different perturbed view of the same underlying image without increasing dataset storage. The following operations were used:

- Random horizontal flip: tf.image.random_flip_left_right;
- Random vertical flip: tf.image.random_flip_up_down;
- Random brightness jitter: tf.image.random_brightness with max_delta = 0.10;
- Random contrast jitter: tf.image.random_contrast with lower = 0.90 and upper = 1.10.

Geometric flips were selected because erythrocyte morphology and intra-cellular parasite structures are not orientation-dependent, and thus flip-based invariance is appropriate. Photometric jitter (brightness/contrast) was included to account for microscope illumination differences, staining intensity variation, and camera exposure changes commonly observed in thin smear imaging.

#### 3.3.5. Dataset Performance Optimizations

To maximize throughput and reduce input latency, the following tf.data optimizations were applied:

- Shuffling: Training samples were randomly permuted using a shuffle buffer of 4096 to mitigate ordering bias and to decorrelate mini-batches.
- Batching: Samples were grouped into mini-batches of 64 for stable gradient estimation and efficient GPU utilization.
- Prefetching: The pipeline used prefetch(tf.data.AUTOTUNE) so that CPU-side preprocessing and GPU-side model execution could overlap, reducing step time. Where applicable, mapping operations were executed with parallel calls (num_parallel_calls = tf.data.AUTOTUNE) to exploit multicore CPU resources.

#### 3.3.6. Separation of Train vs. Evaluation Pipelines

Two dataset variants were constructed: (i) a training pipeline including preprocessing + augmentation + shuffle + batch + prefetch, and (ii) an evaluation pipeline including preprocessing only (no augmentation), followed by batch and prefetch. This separation ensured that reported validation/test metrics reflected performance on unmodified images while still benefiting from augmentation-induced regularization during training.

### 3.4. Network Architectures

Binary classification was formulated with labels y ∈ {0, 1}, where y = 1 denoted Parasitized and y = 0 denoted Uninfected. Each input image was represented as x ∈ R128 × 128 × 3. All networks were implemented using the TensorFlow/Keras functional API and produced a scalar posterior probability $\hat{p}$ = P(y = 1|x) through a sigmoid activation at the output layer. The architectures were designed around parallel feature extraction and feature fusion to combine local texture descriptors with more global contextual representations.

#### 3.4.1. Parallel CNN–Attention–ANN (Primary Model)

The primary model consisted of two parallel branches: (i) a convolutional feature extractor (Branch A) that emphasized local texture and edge statistics, and (ii) a token-based attention pathway (Branch B) that modeled long-range relationships among patch-level representations. For an input x, two feature vectors were produced, zcnn and zattn, which were fused by concatenation and classified by a multilayer perceptron (MLP) head to obtain $\hat{p}$.

z_cnn=φ_cnn(x),    z_attn=φ_attn(x),    z=[z_cnn ; z_attn]

Branch A (CNN feature extractor): A hierarchical convolutional pathway was constructed using repeated convolutional blocks. Each block applied a 2-D convolution followed by batch normalization and a ReLU nonlinearity. Feature depths were increased progressively (32, 64, 128, and 256 filters) to improve representational capacity as spatial resolution decreased. Max-pooling was applied after the first three convolutional stages to reduce spatial size and increase the effective receptive field. A compact global descriptor was obtained using global average pooling (GAP) over spatial dimensions.

$$h^{\left(l\right)}=ReLU(BN(Conv2D(h^{\left(l-1\right)};\;W^{\left(l\right)})))$$

$$z\text{\_}cnn=GAP2D(h^{\left(final\right)})$$

Branch B (token/attention pathway): A patch/token embedding was produced using a strides convolution tokenizer (kernel size 4, stride 4) to generate a low-resolution feature grid, which was reshaped into a token sequence and normalized using layer normalization. Multi-head self-attention (MHSA) was then applied to model long-range interactions among tokens, followed by global average pooling across the token dimension to yield a fixed-length vector.

E=Conv2D{k=4,s=4}x,    T=Reshape(E) ∈ R^(N×d),    T~=LayerNorm(T)

$$Attn(Q,K,V)=softmax((Q\;K^{T})/sqrt(d\text{\_}h))\;V$$

z_attn=GAP1D(MHSA(T~))

Fusion and classifier head: The branch descriptors were fused by concatenation, and the fused representation was passed through an MLP with dropout regularization to reduce overfitting. A final sigmoid unit produced $\hat{p}$, interpreted as the posterior probability of the parasitized class.

$$u_{1}=Dropout\left(0.35\right)\left(ReLU\left(z\;W_{1}+b_{1}\right)\right)$$

$$u_{2}=Dropout(0.25)(ReLU(u_{1}W_{2}+b_{2}))$$

$$\hat{p}=\sigma (u_{2}\;w_{o}+b_{o}),\;\;\;\;\sigma (t)=1/(1+e^{\left(-t\right)})$$

The architectural rationale was to explicitly fuse complementary signals: the CNN branch emphasized local morphology and texture, whereas the attention branch captured global relational structure among patch tokens.

Model 1: Parallel CNN–Attention–ANN Inference

1. Input: x ∈ R^128 × 128 × 3^
2. CNN branch: z_cnn_ ← GAP2D(CNNBlocks(x)).
3. Tokenization: T ← Reshape(Conv2D_k = 4,s = 4_(x)).
4. Attention: T′ ← MHSA(LayerNorm(T)).
5. Attention pooling: z_attn_ ← GAP1D(T′).
6. Fusion: z ← [z_cnn_; z_attn_].
7. Classifier: $\hat{p}$ ← σ(MLP(z)).
8. Output: $\hat{p}$ = P(y = 1|x).

#### 3.4.2. Extended Parallel Variants

To investigate whether additional representational diversity improved discrimination under heterogeneous staining, illumination, and cell morphology, two extended variants were evaluated. Model 2 adopted an expanded multi-branch configuration with additional compact convolutional pathways and a learned gating mechanism to weight branch contributions prior to fusion. In this setting, each branch produced a feature vector zi, and a gating network generated a corresponding scalar weight αi to modulate its contribution before concatenation. Model 3 further increased architectural capacity by incorporating additional attention-style modules and an enhanced fusion head (including expert-like combinations) to improve adaptability across diverse parasite presentations and challenging negative samples. All variants preserved the same binary sigmoid output formulation to ensure direct comparability under identical training and evaluation protocols.

Model 2: Complex Parallel Attention Convolutional Artificial Neural Network

Input: RGB image I of size 128 × 128 × 3

Output: Probability p in [0, 1] for the positive class

1. Preprocess input image I (resize to 128 × 128 if needed, convert to float tensor).
2. Stem feature extraction:
      2.1 X <- Conv-BN-ReLU(I, 32, 3 × 3, stride = 2);
      2.2 X <- Conv-BN-ReLU(X, 32, 3 × 3, stride = 1);
      2.3 X <- Conv-BN-ReLU(X, 64, 3 × 3, stride = 1);
      2.4 X0 <- MaxPool(X, 3 × 3, stride = 2).
3. Residual CNN backbone:
      3.1 Apply residual block(s) at 64 channels using skip Add connections.
      3.2 Downsample to 128 channels with projection shortcut and residual Add.
      3.3 Let F_cnn be the resulting CNN feature map.
4. Channel attention (SE):
      4.1 s <- GlobalAveragePooling(F_cnn);
      4.2 z <- Dense(ReLU)(s); g <- Dense(Sigmoid)(z);
      4.3 g <- Reshape(1, 1, C)(g);
      4.4 F_se <- F_cnn * g.
5. Transformer token branch (from input image):
      5.1 P <- PatchEmbedConv(I, filters = 128, kernel = 8 × 8, stride = 8);
      5.2 T <- Reshape(P) into token sequence;
      5.3 T <- Dropout(T, 0.1);
      5.4 Transformer Block 1: LN -> MHA(4 heads) -> residual -> LN -> MLP(512 -> 128) -> residual;
      5.5 Transformer Block 2: LN -> MHA(4 heads) -> residual -> LN -> MLP(512 -> 128) -> residual;
      5.6 f_tx <- GlobalAveragePooling1D(T).
6. Laplacian/high-frequency branch:
      6.1 L <- Laplacian(I) (Lambda layer);
      6.2 F_lap <- Conv-BN-ReLU stack on L.
7. Inception-like multi-scale branch:
      7.1 From pooled features, compute parallel paths: 1 × 1, 3 × 3, 5 × 5, and pooled + 1 × 1;
      7.2 Concatenate parallel outputs to obtain F_ms.
8. Additional compact/deep CNN branches:
      8.1 Use Conv2D/SeparableConv2D downsampling paths to derive semantic and texture feature maps;
      8.2 Apply another SE-like weighting on one branch (GlobalAvgPool -> Dense -> Dense(sigmoid) -> Multiply).
9. Branch summarization (global pooling):
      9.1 f1 <- GlobalAveragePooling2D(branch A);
      9.2 f2 <- GlobalAveragePooling2D(branch B);
      9.3 f3 <- GlobalAveragePooling1D(transformer branch);
      9.4 f4 <- GlobalAveragePooling2D(branch C).
10. Gate estimation for branch fusion:
      10.1 u <- Concatenate([f1, f2, f3, f4]);
      10.2 h <- Dense(256, ReLU)(u); h <- Dropout(h, 0.2);
      10.3 w <- Dense(4, Softmax)(h) # branch_gates;
      10.4 Split w into scalars [w1, w2, w3, w4] using Lambda layers.
11. Gated fusion:
      11.1 g1 <- w1 * f1; g2 <- w2 * f2; g3 <- w3 * f3; g4 <- w4 * f4;
      11.2 f_fused <- Concatenate([g1, g2, g3, g4]).
12. Classification head:
      12.1 y <- Dense(512, ReLU) -> BN -> Dropout(0.45);
      12.2 y <- Dense(256, ReLU) -> BN -> Dropout(0.35);
      12.3 y <- Dense(96, ReLU) -> Dropout(0.25);
      12.4 p <- Dense(1, Sigmoid)(y).
13. Return p:

Thereby allowing the model to emphasize different feature pathways depending on the input cell appearance.

Model#3 further increased architectural capacity by incorporating additional attention-style modules and an enhanced fusion head (including expert-like combinations) to improve adaptability across diverse parasite presentations and challenging negative samples. Despite architectural differences, all variants preserved the same binary sigmoid output formulation to ensure direct comparability under identical training and evaluation protocols.

### 3.5. Training Configuration

The three evaluated architectures (ResNet50, MobileNet and EfficientNetB0) were not fine-tuned from pretrained backbones in a staged transfer-learning setting; rather, each model was defined as a custom architecture and trained end-to-end from initiali-zation under a matched optimization pipeline. Accordingly, there were no model-specific freezing or unfreezing steps, no partial backbone locking, and no sequential fine-tuning phases that differed across models. Instead, all layers in propose Models#1–#3 and CNN remained trainable throughout training, and fairness was maintained by applying the same core training framework across models, including the same optimizer family (Adam), initial learning rate, stratified 10-fold cross-validation procedure, validation-driven checkpointing, early stopping, and learning-rate reduction on plateau, followed by a final training stage for best-model selection.

All models were trained under a unified optimization protocol to ensure fair comparison across architectural variants. Training was performed using the Adam optimizer, selected for its adaptive moment estimation and stable convergence in deep neural network optimization. The initial learning rate was set to 1 × 10^−3^, and model parameters were updated by minimizing the binary cross-entropy loss, consistent with the binary formulation of parasitized versus uninfected classification. Mini-batch training was conducted with a batch size of 64, balancing gradient stability and GPU memory efficiency. For the 10-fold cross-validation experiments, each fold was trained for up to 10 epochs, whereas the final model used for independent test evaluation was trained for up to 20 epochs to maximize convergence when more training data were available.

To control overfitting and to stabilize training dynamics, a standardized callback strategy was employed, with validation AUC used as the primary model-selection criterion due to its threshold-independent characterization of discriminatory performance. The best-performing weights were saved using ModelCheckpoint, retaining only the epoch that maximized validation AUC. In addition, EarlyStopping was applied with restoration of the best weights to prevent unnecessary training once validation performance ceased. Learning rate scheduling was implemented using ReduceLROnPlateau, which reduced the learning rate by a factor of 0.5 when validation AUC plateaued for 2 consecutive epochs, with a lower bound of 1 × 10^−6^ to avoid excessively small updates. Throughout training and validation, binary accuracy, AUC, precision and recall were computed within the Keras training loop to provide complementary views of overall correctness and class-sensitive behavior.

### 3.6. Experimental Environment

All experiments were conducted in a GPU-enabled deep-learning environment to accelerate training and inference. Model development and training were implemented in TensorFlow (v2.19.0) using the Keras functional API, while input pipelines and augmentation were executed with tf.data to support efficient streaming, shuffling, batching, and prefetching. Dataset partitioning, stratified sampling, and the 10-fold stratified cross-validation protocol were implemented with scikit-learn, which was also used for post-training metric computation (e.g., confusion matrix and classification report) to ensure consistent, reproducible evaluation outside the training loop. In addition, Matplotlib (version 3.10.0) was used to visualize optimization behavior and model discrimination performance, including training/validation learning curves and receiver operating characteristic (ROC) curves. To improve reproducibility, fixed random seeds were applied across Python (version 3.12.12), NumPy (version 2.0.2), and TensorFlow (version 2.19.0), and all experiments were executed under a consistent software configuration throughout training and evaluation.

### 3.7. Evaluation Metrics and Reporting

Model outputs were produced as posterior probabilities of the parasitized class via a sigmoid activation, and probabilities were converted to binary class labels using a fixed decision threshold of 0.5. Classification performance was quantified using standard metrics, including accuracy, precision, recall, and F1-score, which collectively characterized overall correctness and the balance between false positives and false negatives. In addition, discrimination capability across thresholds was assessed using the ROC curve and summarized using the area under the ROC curve (ROC–AUC). For each fold of the stratified cross-validation procedure, predictions were generated for the fold-specific validation subset and all metrics were computed using the corresponding ground-truth labels. Cross-validation outcomes were reported as mean ± standard deviation across folds, thereby reflecting both expected performance and variability due to data partitioning. A reliability diagram evaluates model calibration by comparing predicted probabilities with observed outcomes. A precision-recall (PR) curve assesses classification performance by showing the trade-off between precision and recall across thresholds, particularly in imbalanced datasets.

### 3.8. Hold-Out Test Evaluation

Conventional baseline models were re-run under a harmonized preprocessing and evaluation pipeline, and all comparative tables/narrative statements were audited for consistency after correction. This reduces the risk of implementation asymmetry and ensures that conclusions about comparative performance are based on aligned experimental conditions.

Following cross-validation-based model development, a final model instance was trained on the final training subset and monitored on the internal validation subset for checkpoint selection. The model state achieving the best validation discrimination performance (i.e., the highest validation AUC) was retained and evaluated once on the independent 10% held-out test set, which had been isolated prior to cross-validation to prevent information leakage. Test performance was summarized using the confusion matrix to explicitly report true/false positives and negatives, together with class-wise and aggregate precision, recall, and F1-score obtained from the standard classification report. Finally, ROC curves and the corresponding ROC–AUC values were computed on the test set to provide a threshold-independent measure of separability between parasitized and uninfected cell images.

### 3.9. Comparison with Conventional Models

To contextualize the performance of the proposed architecture against established deep learning baselines, four conventional CNN-based classifiers were implemented and evaluated under a controlled experimental setting. The comparator set comprised (i) a custom CNN developed as a lightweight, task-specific baseline, and three widely adopted canonical CNN backbones: (ii) ResNet50, representing a deeper residual-learning paradigm with strong representational capacity; (iii) MobileNet, reflecting an efficiency-oriented design based on depthwise separable convolutions suitable for re-source-constrained deployment; and (iv) EfficientNetB0, a modern compact architecture derived from compound scaling of network depth, width, and input resolution. All baseline models were trained using an identical training protocol to ensure methodological fairness and to minimize confounding effects arising from differences in optimization or regularization settings. Specifically, the training configuration, including dataset partitioning strategy, preprocessing and normalization pipeline, input size, data augmentation procedures, loss function, optimizer type and hyperparameters (e.g., learning rate schedule, momentum/β parameters), batch size, number of epochs, early stopping criteria, and model-selection rule (e.g., best validation loss or accuracy) was held constant across all networks. Moreover, evaluation was performed using the same validation/testing procedure and decision thresholding scheme, enabling direct, apples-to-apples comparison of predictive performance (e.g., accuracy and class-wise metrics) as well as secondary considerations such as parameter count and computational efficiency. This standardized benchmarking design allows any observed performance differences to be attributed primarily to architectural characteristics rather than variations in training conditions, thereby strengthening the validity of conclusions regarding the relative merits of the proposed model. To assess whether differences between competing models were statistically meaningful, we conducted pairwise Wilcoxon signed-rank tests (two-sided) using these four metrics as paired observations for each comparison. Across all pairwise tests, no comparison reached statistical significance at α = 0.05 (all *p* > 0.05), suggesting that the observed performance differences were not consistently large across metrics. Because the Wilcoxon tests were based on a small number of aggregated observations (n = 4 metrics per model), the results are interpreted as supportive, exploratory evidence, and future work should validate statistical differences using fold-wise repeated experiments or resampling-based inference.

CNN Classification Model

Input: Image X

Output: Predicted class Y

1. X ← Preprocess(X)
2. F1 ← Convolution(X)
3. A1 ← ReLU(F1)
4. P1 ← MaxPooling(A1)
5. F2 ← Convolution(P1)
6. A2 ← ReLU(F2)
7. P2 ← MaxPooling(A2)
8. F3 ← Convolution(P2)
9. A3 ← ReLU(F3)
10. P3 ← MaxPooling(A3)
11. V ← Flatten(P3)
12. D ← Dense(V)
13. D ← ReLU(D)
14. D ← Dropout(D)
15. Y ← Softmax(OutputLayer(D))
16. Return Y

## 4. Results

### 4.1. Cross-Validation Performance on the Training Pool

Performance was first quantified using 10-fold stratified cross-validation on the 90% training pool. Across all evaluated architectures, consistently strong discrimination was observed, with mean ROC–AUC values ≈ 0.99, indicating that the learned representations separated parasitized and uninfected cell images with high reliability under varying fold partitions. Differences among models were primarily reflected in the precision–recall trade-off rather than in ROC–AUC.

**Figure 2:**
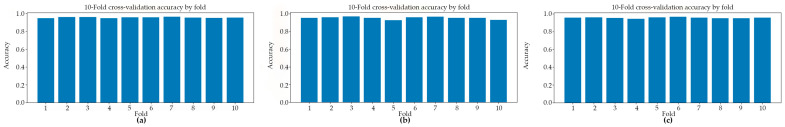
The classification accuracy obtained through 10-fold cross-validation for three distinct models: (**a**) Model#1, (**b**) Model#2, and (**c**) Model#3.

Figure 2 presents the classification accuracy derived from 10-fold cross-validation across three distinct predictive models. The application of 10-fold cross-validation ensures a robust and reliable estimation of model performance by systematically partitioning the dataset into ten equally sized subsets, wherein each subset serves iteratively as the validation set while the remaining folds are employed for training. This methodological approach effectively mitigates the risk of overfitting and reduces variance in performance estimation, thereby providing a more generalized assessment of each model’s classification capability.

**Figure 3:**
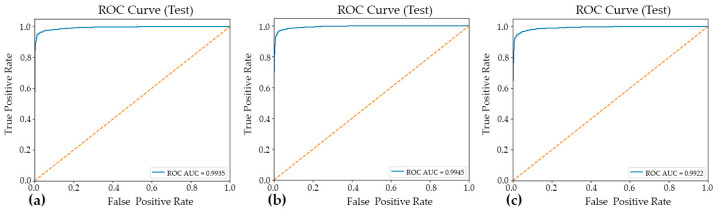
The ROC curves and corresponding AUC–ROC values for the three evaluated models: Model#1 (**a**), Model#2 (**b**), and Model#3 (**c**). The AUC–ROC is reported to summarize each model’s overall discriminative ability across the full range of decision thresholds, facilitating a threshold-independent comparison of classification performance.

Table 1 presents a summary of the fold-aggregated cross-validation performance metrics for all three models, with the corresponding distributions illustrated in Figure 2. Among the evaluated architectures, Model#1 attained the highest overall cross-validation accuracy (0.9567 ± 0.0050), accompanied by well-balanced precision and recall values, indicating consistent and symmetric classification performance across both classes. Model#2 yielded the highest mean precision (0.9808 ± 0.0059); however, this was accompanied by a comparatively reduced recall (0.9258 ± 0.0318), a pattern suggestive of a more conservative decision boundary wherein the model prioritized the minimization of false positives at the expense of an elevated false negative rate. Model#3, by contrast, demonstrated notably high recall (0.9504 ± 0.0132) alongside competitive classification accuracy (0.9558 ± 0.0064) and a similarly strong ROC-AUC score (0.9900 ± 0.0024) (Figure 3), collectively indicating robust sensitivity to parasitized cell patterns throughout the cross-validation procedure. These findings suggest that while all three models achieved strong discriminative performance, each exhibits distinct trade-offs between precision and recall that may carry differing implications depending on the clinical or operational priorities of the intended application.

### 4.2. Convergence Behavior and Training Dynamics

Training curves (accuracy/AUC versus epoch) showed rapid convergence for all models, consistent with the strong separability of the two-class dataset. During final model fitting (up to 20 epochs), the median epoch runtime after the initial compilation overhead was approximately ~11 s/epoch for Model#1 and Model#2, and ~17 s/epoch for Model#3, reflecting the higher computational cost of the most complex parallel variant. Validation AUC-driven checkpointing identified the best-performing epochs and prevented over-training; early stopping was triggered for Model#2 (best epoch = 11) and Model#3 (best epoch = 16), whereas Model#1 continued to the maximum epoch budget and restored the best weights at epoch 20.

### 4.3. Hold-Out Test Performance

After model selection, the best checkpoint from final training was evaluated on the independent 10% hold-out test set (n = 2756) (Table 2). High generalization performance was obtained for Model#1 and Model#2, both achieving approximately 0.97 test accuracy with strong macro-averaged precision, recall, and F1-scores. Model#3 achieved a lower 0.95 test accuracy, driven primarily by a higher rate of false positive predictions for the parasitized class.

### 4.4. Confusion Matrix and Error Profile

**Figure 4:**
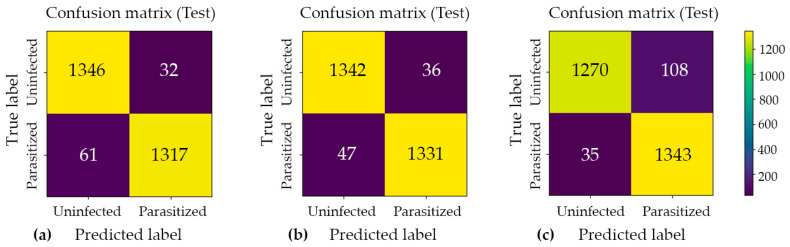
Confusion matrix analysis for the three proposed classification models: (**a**) Model#1, (**b**) Model#2, and (**c**) Model#3. Each matrix illustrates the distribution of predicted versus actual class labels, providing a comprehensive breakdown of true positive, true negative, false positive, and false negative outcomes. The color intensity scale (colorbar) on the right indicates the absolute number of cell crops in each category, where brighter yellow hues represent higher sample counts and darker purple hues represent lower counts. This visualization enables a systematic evaluation of each model’s discriminative performance, particularly in identifying the trade-offs between sensitivity and specificity across the three architectures.

Confusion matrix analysis was employed to examine the distribution of classification errors across two distinct categories: false positives (Type I errors) and false negatives (Type II errors). This approach provides a more granular diagnostic perspective than aggregate accuracy metrics alone. Such analysis is particularly valuable in the context of imbalanced datasets or asymmetric misclassification costs, as it enables the independent evaluation of a model’s sensitivity and specificity. The confusion matrices for Model#1, Model#2, and Model#3, presented in Figure 4, offer a structured decomposition of each model’s predictive performance into four fundamental outcome categories: true positives (TP), true negatives (TN), false positives (FP), and false negatives (FN). By examining the off-diagonal elements of each matrix, which correspond to misclassified instances, meaningful insights can be drawn regarding the nature and directionality of prediction errors specific to each model architecture. A comparative inspection of these three matrices further facilitates an assessment of how each model navigates the inherent trade-off between precision and recall. This evaluation is critical for determining the most appropriate model for the intended application domain.

**Figure 5:**
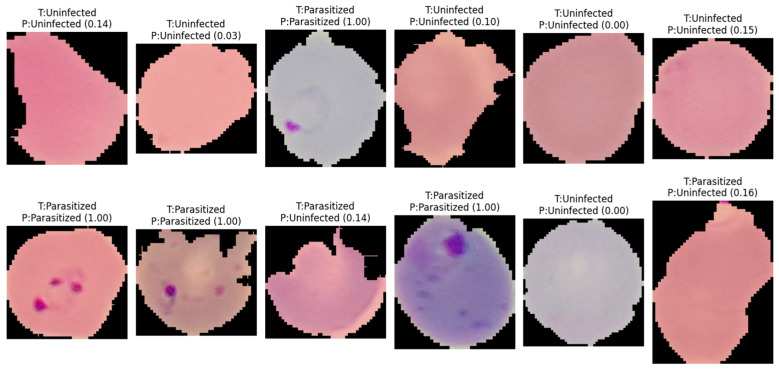
Representative classification outcomes generated by Model#2 on independent test samples. The labels indicate the actual class (T) versus the predicted class (P), with numerical values in parentheses representing the model’s posterior probability (confidence score) for the parasitized class. The results demonstrate the model’s ability to accurately identify ring-form parasites even across varying cell morphologies and staining intensities, as well as its robustness in correctly classifying uninfected erythrocytes with high confidence.

Across all cross-validation folds, the three models achieved near-ceiling ROC–AUC values of approximately 0.99, confirming strong class separability and the overall discriminative capacity of the proposed architectures. However, evaluation on the held-out test set revealed that architectural complexity does not inherently guarantee superior generalization performance. Model#1 and Model#2 yielded the most favorable and stable results, each attaining approximately 0.97 accuracy with low and well-balanced error counts across both classes. Notably, Model#2 achieved the highest classification accuracy among all evaluated architectures, as illustrated in Figure 5, which presents the model’s predictive outputs across the designated categories and confirms its robust generalization to unseen samples. In contrast, Model#3, despite demonstrating heightened sensitivity to parasitized patterns, introduced a substantially elevated false-positive rate under the fixed decision threshold, which consequently reduced its overall test accuracy to approximately 0.95. These findings suggest that optimizing for sensitivity alone, without accounting for the precision–recall trade-off, may compromise overall predictive reliability in binary medical image classification tasks.

**Figure 6:**
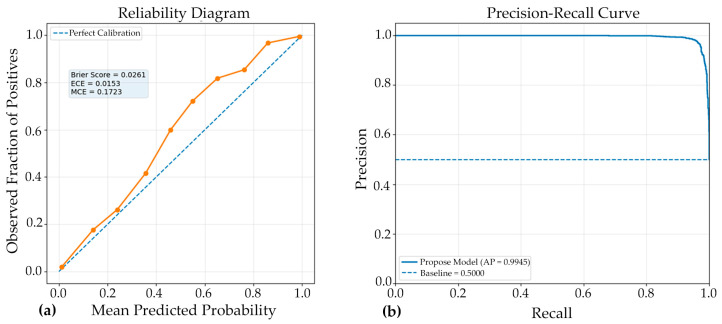
Complementary evaluation of model calibration and discrimination. (**a**) Reliability diagram showing the agreement between predicted probabilities and observed outcomes; closer alignment with the diagonal indicates superior calibration. (**b**) Precision-recall (PR) curve illustrating the trade-off between precision and recall across various classification thresholds. A curve approaching the upper-right corner indicates stronger discriminative performance, especially in scenarios involving class imbalance. Together, these panels demonstrate both the reliability of the predicted probabilities and the model’s robust ability to accurately distinguish positive cases.

The reliability diagram demonstrated that the model achieved good probability calibration (Figure 6), with most predictions lying close to the ideal diagonal line, indicating strong agreement between predicted confidence and observed accuracy. This interpretation is supported by the low Brier score of 0.0261, which reflects high overall probabilistic accuracy and suggests that the predicted probabilities were well aligned with the true outcomes. Likewise, the Expected Calibration Error (ECE) of 0.0153 indicates that, on average, the discrepancy between confidence and actual correctness across all prediction bins was very small, confirming that the Model#2 was generally well calibrated. However, the Maximum Calibration Error (MCE) of 0.1723 reveals that at least one confidence interval exhibited a noticeable local mismatch between predicted probability and empirical accuracy. Thus, while the overall calibration performance was strong and the model can be considered reliable for probability estimation, the presence of a relatively higher MCE suggests that certain confidence regions may still require further calibration refinement.

The PR curve (Figure 6) demonstrated excellent discriminative performance of the proposed Model#2, with an average precision (AP) of 0.9945. This result indicates that the classifier maintained very high precision across a wide range of recall values, reflecting a strong ability to correctly identify positive cases while minimizing false-positive predictions. An AP value this close to 1.0 suggests that the model achieved a highly favorable balance between sensitivity and precision, which is particularly important in medical image classification where both missed detections and incorrect positive identifications can have significant consequences. Overall, the PR curve confirms the robustness and reliability of the model, highlighting its strong suitability for accurate classification performance in the evaluated dataset.

### 4.5. Comparative Performance Analysis with Conventional Models

The classification performance of the four deep learning models (custom CNN, ResNet50, MobileNet, and EfficientNetB0) is compared in Table 3, using accuracy, precision, recall, and F1-score as key evaluation metrics. Overall, the results indicate that the transfer learning–based architectures outperformed the Custom CNN across most metrics. Among all models, MobileNet achieved the highest accuracy (0.9592), recall (0.9481), and F1-score (0.9587), suggesting that it provided the most balanced and effective overall performance for the classification task. EfficientNetB0 showed very similar results, with an accuracy of 0.9590 and an F1-score of 0.9585, indicating that its predictive capability was nearly equivalent to that of MobileNet. ResNet50 demonstrated the highest precision (0.9863), which implies that it was the most effective model in minimizing false-positive predictions; however, its recall (0.9183) was lower than that of MobileNet and EfficientNetB0, suggesting comparatively weaker sensitivity in identifying true positive cases. In contrast, the custom CNN produced the lowest values across all major metrics, including accuracy (0.9419), recall (0.9077), and F1-score (0.9398), indicating inferior performance relative to the pretrained models. Taken together, these findings suggest that lightweight pretrained architectures, particularly MobileNet and EfficientNetB0, provided superior and more balanced classification performance, while ResNet50 may be preferred in contexts where precision is of primary importance. The reported variation values further reflect differences in performance stability across models, although these should be interpreted carefully depending on the number of experimental runs or folds used in the evaluation.

To examine whether the observed differences in predictive performance among the evaluated models were statistically meaningful, pairwise two-sided Wilcoxon signed-rank tests were conducted. The analysis utilized the reported performance indicators—accuracy, precision, recall, and F1-score—as paired observations for each model comparison. The Wilcoxon signed-rank test was selected as a non-parametric alternative to parametric paired tests, as it does not require the assumption of normality and is well suited for paired comparative analysis. The results showed that none of the pairwise comparisons reached statistical significance at the 0.05 level (all *p* > 0.05). These findings suggest that, based on the specific set of summary performance metrics considered, the apparent differences between models may not be sufficiently large or consistent to support a statistically significant performance advantage of one model over another.

However, these results should be interpreted with caution. Specifically, the Wilcoxon test in this context was performed on a very limited number of paired observations (n = 4), corresponding only to the four aggregated performance metrics rather than fold-wise results, repeated experimental runs, or sample-level prediction outcomes. Consequently, the statistical power of the test is limited, and the analysis should be regarded as an exploratory supplementary comparison rather than definitive inferential evidence. Future studies should incorporate repeated cross-validation or multiple independent runs to provide a more robust basis for statistical significance testing.

## 5. Discussion

In this study, the proposed malaria cell-classification pipeline achieved strong discriminative performance. Cross-validation results indicated stable generalization, and the hold-out test confirmed that the final checkpoint selected by validation AUC transferred well to unseen samples. These findings support the utility of combining convolutional feature extractors with attention-based aggregation for binary discrimination between parasitized and uninfected red blood cells, particularly when microscopy artifacts and staining variability are present.

### 5.1. Image Variation in NIH–NLM Dataset

The absence of statistically significant differences among models should also be interpreted in light of image variation within the dataset, which can substantially influence both absolute performance and the stability of model rankings. Microscopy images often exhibit heterogeneous acquisition conditions (e.g., illumination non-uniformity, staining intensity, focus drift, sensor noise, and background debris), as well as biological and morphological variability (e.g., size and shape differences, partial occlusion, orientation changes, and inter-sample heterogeneity). Such variability can introduce domain shift within the dataset, increasing overlap between class-conditional feature distributions and thereby reducing the margin by which any single architecture consistently outperforms others. Consequently, models may achieve similar aggregate metrics while relying on different inductive biases and failure modes, leading to performance fluctuations that are not uniformly expressed across accuracy, precision, recall, and F1-score.

### 5.2. Comparison with Recent High-Performance Architectures on the NIH–NLM Dataset

Recent studies on the NIH-NLM malaria dataset have reported comparable or slightly higher accuracy, often by leveraging modern backbones with additional multi-scale or residual–dense modules. For example, the EfficientNetB2-based Dense Residual Inception (EDRI) model reported 97.68% accuracy with AUC = 99.76%, alongside high precision and F1-score, outperforming several baseline ImageNet backbones under the same experimental framework [32]. Notably, the EDRI ablation analysis suggested that combining residual, dense, and inception components contributed synergistically to the final performance, indicating that multi-path feature reuse and multi-scale representations are beneficial for malaria cell morphology classification. The outcomes of the present work were consistent with this direction: the observed AUC values (reported in the Section 4) suggest that attention-based integration can similarly enhance separability by emphasizing diagnostically relevant regions, especially when parasite appearance is subtle. Differences in reported accuracy across studies are likely attributable to architectural capacity, the exact data splitting policy, and augmentation intensity. The EDRI study explicitly highlighted that misclassifications often arose from staining interference, blurred focus, or low-contrast regions, which aligns with common failure modes in microscopy-based recognition and supports the interpretation that residual errors in the present work are plausibly driven by similar image-quality constraints [32].

### 5.3. Ensembles and Cross-Validation Rigor

A key trend in the literature is the use of stronger validation protocols and ensemble aggregation to reduce variance. An EfficientNetB0-based approach using stratified 10-fold cross-validation reported an average accuracy near 97.56% across folds [33]. When an ensemble was formed from the fold-specific models, performance increased to 98.29% accuracy, AUC = 99.76%, and F1-score = 98.28%, indicating that ensembling can meaningfully improve robustness even in a relatively low-variability binary microscopy task. That study further reported an external validation phase using a held-out dataset not previously used in training/testing to reduce overfitting concerns. In the present work, fold-wise evaluation (as detailed in Section 3 and Section 4) similarly reduced dependence on a single split. However, the literature suggests that further improvements might be realized by (i) explicitly building an ensemble across folds and (ii) validating on an additional “never-seen” subset from a distinct acquisition batch or site, which would better approximate deployment conditions and quantify domain-shift sensitivity.

### 5.4. Efficiency-Oriented Designs for Deployment

Beyond achieving peak accuracy, several studies emphasize the necessity of deployability within resource-constrained environments. A compact system integrating advanced augmentation strategies and compression-oriented learning reported test accuracies reaching 0.9950 for 28 × 28 images and approximately 0.9923 for 32 × 32 images. This system required only about 4600 Floating Point Operations (FLOPs) and achieved inference times of less than one second on smartphones, which highlights the practical feasibility of real-time screening on portable devices [3]. Such results underscore a critical trade-off between architectural complexity and operational efficiency. While high-capacity architectures, including multi-branch and attention-based designs, can significantly improve representational power, lightweight designs may be more appropriate in settings where throughput and energy constraints are the dominant factors. Consequently, a practical extension of the present work would involve the knowledge distillation of the attention-augmented model into a smaller student network. This approach could potentially combine the superior discrimination benefits observed in this study with the deployment advantages demonstrated in previous efficiency-centered frameworks [3].

### 5.5. Consistency with Earlier CNN Evidence and the Role of Transfer Learning

Earlier work demonstrated that strong performance can be achieved with carefully designed CNNs on single-cell images. A 16-layer CNN reported 97.37% average accuracy under 10-fold cross-validation on 27,578 single-cell images, substantially outperforming a transfer-learning baseline trained on the same data [4]. These findings support the broader observation that malaria cell classification can benefit from task-specific feature learning rather than relying solely on generic ImageNet features, although modern transfer learning often becomes competitive when paired with appropriate fine-tuning and augmentation. At the same time, transfer-learning-based comparisons need to be interpreted carefully because experimental protocols can substantially alter reported metrics. A separate study using multiple standard CNN architectures (AlexNet/GoogLeNet/ResNet/VGG) applied augmentation and an 80/20 split and presented qualitative prediction examples described as achieving 100% accuracy on illustrated samples [34]. Because such reporting is not directly equivalent to full test-set accuracy under controlled evaluation, it reinforces the importance of standardized reporting (confusion matrices, ROC–AUC, and cross-validated statistics), which was addressed in the present work.

### 5.6. Implications of Split Strategy and Statistical Validation

A persistent concern in medical image classification is the risk of optimistic performance estimates when images from the same subject are distributed across both training and testing partitions. Rajaraman et al. explicitly demonstrated that patient-level cross-validation produced a lower accuracy of 0.959 compared to 0.986 obtained through random splitting on the same task. This discrepancy indicates that data leakage can materially inflate performance metrics if not strictly controlled [12]. Furthermore, the researchers employed statistical testing to compare model variants, reporting significant differences in accuracy and AUC through the application of χ^2^ tests with *p*-values on the order of 10^−3^ to 10^−2^ [32]. Accordingly, while the present results indicate strong classification performance, the most conservative interpretation is that the reported metrics apply specifically to the image-level split and the experimental settings described in the methodology. For translation toward clinical-grade validation, future work should incorporate patient-level or slide-level grouping during the splitting process, provided that the necessary metadata are available. Additionally, applying rigorous statistical comparisons across competing models and validation folds is essential to quantify whether the observed gains over baseline architectures are statistically significant and repeatable under diverse clinical conditions.

### 5.7. Limitations and Future Directions

Although the high ROC–AUC values achieved in this study suggest robust separability, the remaining misclassifications likely arise from microscopy-related factors that obscure discriminative signals. These factors include staining artifacts, focus variation, and low contrast, which are limitations also reported in prior literature [32]. Based on these observations, three methodological extensions are well motivated by the existing body of research. First, external validation on a dataset entirely independent of the training and testing phases is necessary to better characterize domain shift, as seen in ensemble-based evaluation studies [33]. Second, model compression or knowledge distillation should be explored to improve throughput and portability while preserving classification performance. This follows efficiency-centered frameworks that have successfully demonstrated smartphone-capable inference for field deployment [32]. Finally, future investigations should adopt patient-level or slide-level splitting strategies combined with rigorous statistical testing. This is essential because the split strategy can meaningfully alter reported accuracy, and statistical comparisons are required to clarify whether the observed improvements are robust and repeatable [12].

The statistical comparison provides additional context for interpreting the performance rankings across models. Pairwise Wilcoxon signed-rank tests (two-sided), performed using the four-summary metrics (accuracy, precision, recall, and F1-score) as paired observations, did not identify statistically significant differences between any model pairs at α = 0.05 (all *p* > 0.05). This result suggests that the observed variations in performance are modest and not uniformly consistent across metrics, indicating that several models achieve broadly comparable predictive behavior under the evaluated setting. Nevertheless, because the test was applied to a small set of aggregated metrics (n = 4), these findings should be viewed as supportive rather than definitive; a more rigorous assessment would require repeated cross-validation or multiple independent runs to enable fold-level statistical inference and improve power.

The reliability diagram indicates that the model was well calibrated overall, as the predicted probabilities were closely aligned with the observed frequencies across most confidence bins. This interpretation is supported by the low Brier score (0.0261) and ECE (0.0153), which together suggest strong probabilistic accuracy and only minimal average miscalibration. Nevertheless, the MCE value of 0.1723 shows that a larger calibration gap was still present in at least one interval, implying that some localized overconfidence or underconfidence remained. Thus, while the model demonstrates reliable confidence estimation in general, further calibration improvement may still be beneficial for reducing isolated prediction inconsistencies.

Overall, the present work aligns with and complements recent literature indicating that multi-path feature learning or attention mechanisms can significantly improve discrimination. Furthermore, the findings reinforce that rigorous validation protocols and ensembling can boost model stability, while practical deployment considerations require explicit attention to computational cost to ensure viability in real-world clinical settings.

## 6. Conclusions

In this study, a binary malaria cell-image classification framework was developed and evaluated using a publicly available thin blood smear dataset consisting of 27,558 images. Parallel deep architectures were implemented to fuse complementary representations, effectively combining convolutional feature extraction with attention-based token modeling. Model performance was rigorously assessed through a 10-fold stratified cross-validation protocol and a strictly independent 10% hold-out test set to ensure a reliable estimate of generalization.

Across the cross-validation phase, all three architectures demonstrated consistently strong discriminative performance, achieving mean accuracies between 0.9538 and 0.9567 and mean ROC–AUC values of approximately 0.989 to 0.990. These results indicate that parasitized and uninfected cell crops remain reliably separable under repeated resampling. On the independent test set, the primary parallel CNN–attention model (Model#1) and the extended parallel model (Model#2) achieved the highest generalization, both reaching an accuracy and macro F1-score of 0.97. Specifically, Model#2 produced fewer false negatives (47) compared to Model#1 (61) while maintaining comparable false positive rates, which suggests an improved sensitivity–specificity balance. In contrast, the most complex variant, Model#3, achieved higher sensitivity with only 35 false negatives but incurred a substantial increase in false positives (108), thereby reducing the overall test accuracy to 0.95. These findings demonstrate that increasing architectural complexity does not necessarily improve generalization and that moderate-capacity parallel fusion provides the most stable performance for this dataset.

Overall, the results support the effectiveness of parallel feature fusion for malaria cell-image classification and highlight the importance of evaluating error profiles beyond aggregate metrics when considering deployment in screening workflows. Future work should extend evaluation to slide-level or patient-level partitioning and incorporate external validation across various staining and acquisition conditions. Furthermore, investigating threshold optimization and probability calibration would allow for tailoring operating points to specific clinical requirements. Integrating this classification framework with detection and segmentation modules for full-field smear images, alongside expanding to multi-stage parasite classification, would ultimately provide a more clinically comprehensive diagnostic pipeline.

Notwithstanding these promising results, the present framework should be interpreted as a cell-level decision-support approach rather than a clinically deployable standalone diagnostic system. Several limitations constrain immediate clinical use. First, the study relied on a publicly available dataset of cropped single-cell images, whereas routine malaria diagnosis is performed on full-slide smears requiring parasite localization across large fields of view, estimation of parasitemia, and integration with laboratory quality-control procedures. Second, because slide-level and patient-level metadata were unavailable, validation was performed at the image level; as noted in the literature and in our discussion, such a design may overestimate real-world generalization relative to grouped partitioning strategies. Third, residual model errors are likely influenced by microscopy-related confounders, including staining variability, focus drift, illumination differences, and low-contrast parasite morphology. Finally, practical implementation in point-of-care or resource-limited settings would require additional attention to calibration, interpretability, computational efficiency, and external validation across heterogeneous acquisition environments. Accordingly, further work should focus on slide-level detection pipelines, patient-/slide-level validation, multi-site prospective testing, and deployment-aware optimization before this framework can be considered for clinical adoption.

## Figures and Tables

**Table 1:** 10-Fold cross-validation results (training pool, mean ± standard deviation).

Model	Accuracy	Precision	Recall	F1-Score	ROC-AUC
Model#1	0.9567 ± 0.0050	0.9660 ± 0.0110	0.9469 ± 0.0135	0.9563 ± 0.0052	0.9891 ± 0.0017
Model#2	0.9538 ± 0.0145	0.9808 ± 0.0059	0.9258 ± 0.0318	0.9522 ± 0.0160	0.9903 ± 0.0036
Model#3	0.9558 ± 0.0064	0.9611 ± 0.0147	0.9504 ± 0.0132	0.9556 ± 0.0063	0.9900 ± 0.0024

**Table 2:** Hold-out test set performance.

Model	Accuracy	Macro Precision	Macro Recall	Macro F1
Model#1	0.9663	0.9768	0.9566	0.9666
Model#2	0.9697	0.9739	0.9662	0.9700
Model#3	0.9481	0.9216	0.9732	0.9467

**Table 3:** Comparative performance of custom CNN, ResNet50, MobileNet, and EfficientNetB0 expressed as mean ± standard deviation across evaluation metrics.

Model	Accuracy	Precision	Recall	F1-Score
Custom CNN	0.9419 ± 2.4302	0.9743 ± 1.1929	0.9077 ± 1.1011	0.9398 ± 2.5160
ResNet50	0.9528 ± 0.0100	0.9863 ± 1.2561	0.9183 ± 0.0245	0.9509 ± 0.0100
MobileNet	0.9592 ± 2.4403	0.9697 ± 1.7803	0.9481 ± 2.9695	0.9587 ± 2.7152
EfficientNetB0	0.9590 ± 2.5575	0.9717 ± 2.6483	0.9458 ± 0.0100	0.9585 ± 3.0644

## Data Availability

The codes are available at the following GitHub repository: https://github.com/Pongphan/Ensemble-Parallel-Neural-Networks (accessed on 25 January 2026).
